# Relationship between genome and epigenome - challenges and requirements for future research

**DOI:** 10.1186/1471-2164-15-487

**Published:** 2014-06-18

**Authors:** Geneviève Almouzni, Lucia Altucci, Bruno Amati, Neil Ashley, David Baulcombe, Nathalie Beaujean, Christoph Bock, Erik Bongcam-Rudloff, Jean Bousquet, Sigurd Braun, Brigitte Bressac-de Paillerets, Marion Bussemakers, Laura Clarke, Ana Conesa, Xavier Estivill, Alireza Fazeli, Neža Grgurević, Ivo Gut, Bastiaan T Heijmans, Sylvie Hermouet, Jeanine Houwing–Duistermaat, Ilaria Iacobucci, Janez Ilaš, Raju Kandimalla, Susanne Krauss-Etschmann, Paul Lasko, Sören Lehmann, Anders Lindroth, Gregor Majdič, Eric Marcotte, Giovanni Martinelli, Nadine Martinet, Eric Meyer, Cristina Miceli, Ken Mills, Maria Moreno-Villanueva, Ghislaine Morvan, Dörthe Nickel, Beate Niesler, Mariusz Nowacki, Jacek Nowak, Stephan Ossowski, Mattia Pelizzola, Roland Pochet, Uroš Potočnik, Magdalena Radwanska, Jeroen Raes, Magnus Rattray, Mark D Robinson, Bernard Roelen, Sascha Sauer, Dieter Schinzer, Eline Slagboom, Tim Spector, Hendrik G Stunnenberg, Ekaterini Tiligada, Maria-Elena Torres-Padilla, Roula Tsonaka, Ann Van Soom, Melita Vidaković, Martin Widschwendter

**Affiliations:** Institut Curie – Research Center, UMR3664 CNRS/IC, 26 rue d’Ulm, Paris cedex 05, F-75248 France; Seconda Università degli Studi di Napoli, Naples, IT, Italy; Istituto Italiano di Tecnologia (IIT), Milan, IT, Italy; Istituto Europeo di Oncologia (IEO), Milan, IT, Italy; University of Oxford, Oxford, UK; Cambridge University, Cambridge, UK; INRA, UMR 1198 Biologie du Développement et Reproduction, Jouy-en-Josas, FR F-78350 France; CeMM Research Center for Molecular Medicine of the Austrian Academy of Sciences, Vienna, AT, Austria; Swedish University of Agricultural Sciences, Uppsala, SE, Sweden; University of Montpellier, Montpellier, FR, France; Ludwig Maximilians University of Munich, Munich, DE, Germany; Gustave-Roussy, Villejuif, FR, France; Radboud University Nijmegen, Nijmegen, NL, Netherlands; European Molecular Biology Laboratory, European Bioinformatics Institute, Hinxton, Cambridge, UK; Centro de Investigación Príncipe Felipe, Valencia, ES, Spain; Centre for Genomic Regulation (CRG), Barcelona, Spain and Universitat Pompeu Fabra (UPF), Barcelona, Spain; University of Sheffield, Sheffield, UK; Center for Animal Genomics, Institute of physiology, Veterinary Faculty, University of Ljubljana and Medical school, University of Maribor, Ljubljana, Slovenia; Centre Nacional d’Anàlisi Genòmica, Barcelona, ES, Spain; Leiden University Medical Center, Leiden, NL, Netherlands; Centre Hospitalier Universitaire, Nantes, FR, France; University of Bologna, Bologna, IT, Italy; Department of Women’s Cancer, UCL Elizabeth Garrett Anderson Institute for Women’s Health, University College London, 74 Huntley Street, London, WC1E 6AU UK; Helmholtz Center, Munich, DE, Germany; Department of Biology, McGill University, Montreal, QC Canada; Karolinska Institute, Stockholm, SE, Sweden; German Cancer Research Centre, Heidelberg, DE, Germany; Canadian Institutes of Health Research, Ottawa, CA Canada; Institut de Chimie, UMR CNRS 7272/UNSA, Nice, FR, France; Centre National de la Recherche Scientifique, Paris, FR, France; University of Camerino, Camerino, IT, Italy; Queen’s University Belfast, Belfast, UK; University of Konstanz, Konstanz, DE, Germany; CNRS UMR7221, Museum National d’Histoire Naturelle, Paris, FR, France; Universitäts Klinikum Heidelberg, Heidelberg, DE, Germany; University of Bern, Bern, CH, Switzerland; Institute of Biochemistry and Biophysics, PAS, Warsaw, PL, Poland; Universite Libre de Bruxelles, Bruxelles, BE, Belgium; University of Maribor, Maribor, SI, Slovenia; Science Europe, Brussel, Europe, BE, Belgium; Vrije Universiteit Brussel, Brussel, BE, Belgium; Katholieke Universiteit Leuven, Leuven, BE, Belgium; Vlaams Instituut voor Biotechnologie, Gent, BE, Belgium; University of Manchester, Manchester, UK; University of Zurich, Zurich, CH, Switzerland; Utrecht University, Utrecht NL, The Netherlands; Max-Planck-Institute for Molecular Genetics, Berlin, DE, Germany; University of Magdeburg, Magdeburg, DE, Germany; Kings College London, London, UK; Medical School University of Athens, Athens, GR, Greece; Institut de Génétique et de Biologie Molécularie et Cellulaire, Strasbourg, FR, France; University of Gent, Gent, BE, Belgium; Institute for Biological Research, Belgrade, RS, Serbia

**Keywords:** Genome, Epigenome, Microbiome, Environment

## Abstract

Understanding the links between genetic, epigenetic and non-genetic factors throughout the lifespan and across generations and their role in disease susceptibility and disease progression offer entirely new avenues and solutions to major problems in our society. To overcome the numerous challenges, we have come up with nine major conclusions to set the vision for future policies and research agendas at the European level.

The Human Genome Project was completed in 2003 and led to the identification of all human genes. However, the fundamental question that remains unanswered is how do genes function and how are they regulated? Epigenetics may provide many crucial answers. Epigenetics encompasses all processes that lead to heritable changes in gene expression as cells divide, while epigenomics refers to analysis of epigenetic changes across the whole genome in a cell or entire organism [1, 2]. Typically, in a multi-cellular organism, each cell type will be characterised by the same genome, along with as many epigenomes as there are distinct cell types. Epigenetics combined with genetics is a rapidly growing field with promising implications for health and disease because many common diseases result from the interplay between the genetic make-up of individuals and the environmental factors to which they are exposed [3]. Currently, however, there is limited knowledge on the combined role of genetic and non-genetic factors thus hampering personalised medicine. A conceptual goal is to identify a cascade of genetic/epigenetic factors that underlie the development of chronic diseases. For example, a number of candidate genes have been associated with irritable bowel syndrome, but little research has examined the mechanistic impact on epigenetics [4]. Likewise, even though environmental factors such as stress, life-style, nutrition, air pollution and infections lead to allergies, the genetic and epigenetic contributions are not well understood [5, 6].

The reversible nature of epigenetic changes has attracted interest in exploring their potential as targets for the development of novel and more individualised medical treatments.

Europe, with additional effort from Member States, is showing leadership in the field of epigenetics and epigenomics and more than €200 Million were invested in research projects and infrastructure through Framework Programmes 6 and 7 (Table 1). For example, the BLUEPRINT project is focusing on distinct types of haematopoietic cells from healthy individuals and their malignant leukaemic counterparts with the aim of generating at least 100 reference epigenomes and studying them to advance and exploit knowledge of the underlying biological processes and mechanisms in health and disease [7].

**Table 1:** **FP7 Cooperation projects and network of excellence that were represented at the workshop**

Acronym	Project description	Website
ATLAS	Development of Laser-Based Technologies and Prototype Instruments for Genome-Wide Chromatin ImmunoPrecipitation Analyses	http://www.atlas-eu.com/
BLUEPRINT	A BLUEPRINT of haematopoietic Epigenomes	http://www.blueprint-epigenome.eu/
CANCERDIP	The use of Methylated DNA Immunoprecipitation MeDIP in cancer for better clinical management	http://www.cancerdip.eu/
CELLOMATIC	High Throughput Systematic Single Cell Genomics using Micro/Nano-Fluidic Chips for Extracting, Pre-analysing, Selecting and Preparing Sequence-ready DNA	http://www.cellomatic.eu/
CURELUNG	Epigenetic therapeutic strategies for improving lung cancer diagnosis	http://www.curelung.eu/
ELIXIR	European Life-Science Infrastructure	http://www.elixir-europe.org/about
EPIFEMCARE	Epigenetics for Female Personalised Cancer Care	http://www.epifemcare.eu/
EPIGENESYS	Epigenetics towards systems biology	http://www.epigenesys.eu/
ESGI	European Sequencing and Genotyping Infrastructure	http://www.esgi-infrastructure.eu/
EUROBATS	Identifying biomarkers of ageing using whole transcriptome sequencing	http://www.eurobats.eu/
GENCODYS	Genetic and Epigenetic Networks in Cognitive Dysfunction	http://www.gencodys.eu/index.php
GENICA	Genomic instability in cancer and pre-cancer	http://genica.unige.ch/
GEUVADIS	Genetic European Variation in Disease	http://www.geuvadis.org/
IDEAL	Integrated research on developmental determinants of Aging and Longevity	http://www.ideal-ageing.eu/
MARK-AGE	European study to establish biomarkers for human aging	http://www.mark-age.eu/
MEDALL	Mechanisms of the Development of ALLergy	http://medall-fp7.eu/
MODHEP	An integrative genomic-epigenomic approach to liver cancer	http://www.modhep.eu/
NGS-PTL	Next Generation Sequencing platform for targeted Personalized Therapy of Leukemia	http://www.ngs-ptl.com/
RADIANT	Rapid development and distribution of statistical tools for high-throughput sequencing data	http://www.radiant-project.eu/
READNA	REvolutionary Approaches and Devices for Nucleic Acid Analysis	http://www.cng.fr/READNA/
SETTREND	Schistosoma epigenetics: targets, regulation, new drugs	http://settrend.cebio.org/
SIROCCO	Silencing RNAs: organisers and coordinators of complexity in eukaryotic organisms	http://www.sirocco-project.eu/
SWITCHBOX	Homeostatic mechanisms to facilitate maintenance of health from early life through to aging	http://www.switchbox-online.eu/
**International consortia**		
IHEC	International Human Epigenome Consortium	http://www.ihec-epigenomes.org/
**Cost actions**		
TD0905 Epigenetics from bench to bedside		http://www.cost.eu/domains_actions/cmst/Actions/TD0905
COST- FA1201– Epigenetics and periconception environment		http://www.cost.eu/domains_actions/fa/Actions/FA1201
COST-BM– 1201 Developmental origins of chronic lung diseases		http://www.cost.eu/domains_actions/bmbs/Actions/BM1201
COST- BM1102 Ciliates as model systems to study genome evolution, mechanisms of non-Mendelian inheritance, and their roles in environmental adaptation		http://www.cost.eu/domains_actions/bmbs/Actions/BM1102
COST Action BM1106 ‘The Genes in Irritable Bowel Syndrome Research Network Europe (GENIEUR)’		http://www.cost.eu/domains_actions/bmbs/Actions/BM1106
COST-BM1007 – Mast cells and basophils – targets for innovative therapies		http://www.cost.eu/domains_actions/bmbs/Actions/BM1007
BM1006 Next Generation Sequencing Data Analysis network (SeqAhead)		http://www.cost.eu/domains_actions/bmbs/Actions/BM1006
BM0806 - Recent advances in histamine receptor H4R research		http://www.cost.eu/domains_actions/bmbs/Actions/BM0806
BM0801 Translating Genomic and epigenetic Studies of MDS and AML (EUGESMA)		http://www.cost.eu/domains_actions/bmbs/Actions/BM0801

With this aim, the European Commission's Directorate General for Research and Innovation (DG RTD) and Cooperation in Science and Technology (COST) organised a joint strategic workshop “Relationship between genome and epigenome”. The workshop addressed the links between genetic, epigenetic and non-genetic factors throughout the lifespan and across generations, their role in health and disease including disease susceptibility and progression, and the associated challenges of data handling/storage and interpretation. The outcomes of the workshop will inform future research priorities and are summarised in Figure 1.

**Figure 1:**
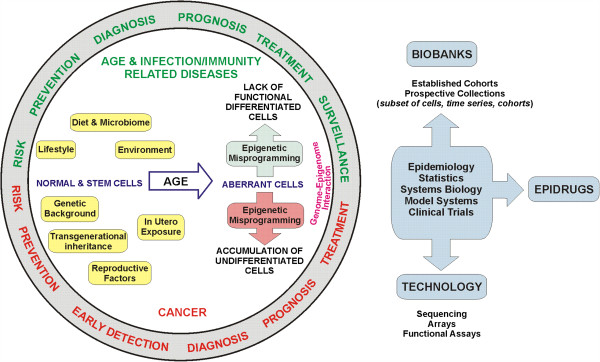
**Understanding the relationship between genome and epigenome and their role in health and disease enables the development of tools for personalized medicine including risk prediction, disease prevention and treatment.** The EU funding provides a platform, enables collaborative work and facilitates to achieve the set aims in order to consolidate Europe's leadership position in Epigenetics.

Major issues for future research include the following points:

1. In order to identify good surrogate epigenomic marks that would corroborate the influence of environmental exposure on the epigenome (including periconception environment, lifestyle, reproductive factors, microbiome etc.) and allow for the prediction and prevention of the development of chronic diseases, detailed research in humans and model organisms and careful sample acquisition (more tissue and cell specific epigenomes, time series, epigenomic variation etc.) is required. Parental conditions before, during and after conception (periconception period) may induce epigenetic changes in gametes and embryos [8]. Such changes may adversely affect the offsprings’ future health, development, productivity and fertility [3]. The connection between the perinatal factors and later outcomes in life was illustrated by describing the relationship between birth weight and incidence of diseases in older age such as cardiac disease [9]. Studies of historical famines already yielded key evidence for the association of early life environmental exposure and differences in the adult epigenome [10]. Like the field itself, these studies are in their infancy and ongoing genome-wide studies are expected to result in the identification of epigenetic alterations that are triggered by non-genetic factors leading to particular disease phenotype. The microbiome has strong parallels with the epigenome in that it is complex and may reflect environmental exposure (of the host from which the micobiome was obtained) and might also impact on how non-genetic factors lead to epigenetic changes (i.e. by modulating hormonal levels [11]). Accumulating data demonstrate a crucial impact of the microbiome on health and disease.
2. With the increase of chronological age, an increase of gene promoter methylation paralleled by global hypomethylation across the genome can be observed. This is remarkably similar to the DNA methylation changes seen in cancer [12] suggesting that similar underlying mechanisms may be involved. More age-stratified data are required to understand the relationship between the epigenome, the microbiome and the environment during the course of life and its impact on allergy and chronic diseases.
3. The genome-epigenome interaction is also crucially involved in the biology, character and extent of an established disease and not just in disease development. This is reflected for instance in the role that the chromatin and epigenome plays in DNA damage repair [13]. Epigenetic markers allow for the prediction of the natural behaviour of a disease (prognostic markers) and the likelihood of responding to a specific treatment (predictive markers). Testing and validating these markers in clinical trials and benchmarking against established strategies will be crucial in order to improve disease outcome.
4. Studies of the effects and the mechanistic impact of epidrugs (drugs that can effect epigenomic modifications) and their impact on the genome, development and validation of new epigenetic drug candidates and rational design of combination therapies of genetic and epigenetic drugs should be encouraged to cure diseases or at least improve the efficacy of current treatment modalities as recently demonstrated [14]. Structural and functional information from chromatin and DNA modifying enzymes and the development of small molecules active on specific epi-targets are crucial for the development of new therapeutic approaches. Epigenetic therapy tries to reverse such aberrations following disruption of the epigenetic signal balance through the use of both natural compounds and synthetic molecules [15]. For instance, pharmacological inhibition of EZH2 (enhancer of zeste homolog 2, a Histone-lysine N-methyltransferase) was recently shown as a promising new tool with which to treat cancer [16]. Many clinical trials are already ongoing, and epigenetic therapy (azacytidine) has recently been approved by the United States Food and Drug Administration (US FDA) for use in the treatment of Myelodysplastic Syndrome (MDS) and Primary Cutaneous T-cell Lymphoma (CTCL) [17].
5. Studies to identify functional relationships between epigenetics and genetics require analysis of ex vivo samples of primary cells, and therefore the sampling, sorting and analytical procedures need to be optimised and adapted. Cell heterogeneity (variation among cells) is a challenge in gaining a thorough understanding of genome status, gene expression and the role of underlying epigenetic mechanisms. This is true for many cellular processes, such as genome remodeling during reprogramming or the conversion of somatic cells to pluripotent cells. Therefore collecting the most appropriate samples in order to address a specific set of questions and miniaturization of technologies for the analyses of single cells [18, 19] is crucial.
6. Epigenomic and genomic data sets are complex and multi-dimensional, and their interpretation requires the further development of data analysis tools/software. A large amount of data has already been acquired and is highly multidimensional and multimodal; therefore it is the analysis that remains the challenge. DNA and chromatin exist in a 3D space. Transcriptome data are complex: all transcripts, including non-coding (nc) RNAs, overlap other transcripts and quantification is not trivial. Performing data analysis by integrating data from different repositories (some of which are difficult to find) is problematic because of the different methodologies used to acquire the data sets [20]. There is a need to establish robust benchmarks for data analysis for the comparison of different analytical approaches/software.
7. Integrating the findings from -omics research into clinical practice is one of the major challenges of the future. Systems biology approaches are advantageous in providing predictive models of associations between epigenomic/genomic data and phenotypes offering an entry point for assays into functional relationships. Understanding the functional/mechanistic role of epigenetic marks is highly desirable, but that in many cases it may be difficult to directly obtain such insight. Systems biological approaches could identify predictive models from multi-modal data to support associations that can then be tested in functional models.
8. Improved collaborations should be fostered by the establishment and harmonization of standard operating procedures for sample processing, data acquisition and formatting; and by the development of software that is user-friendly for the non-specialist as well as facilitating an Open Access policy to allow free data sharing and automatic mining of publications. Current European effort should be aligned with those of the International Human Epigenome Consortium (http://www.ihec-epigenomes.org/) coordinating epigenome mapping and characterisation worldwide to avoid redundant research effort, to implement high data quality standards, to coordinate data storage, management and analysis and to provide free access to the epigenomes produced.
9. European Union (EU) consortia and COST Actions have tremendously shaped and consolidated Europe’s leadership position in Epigenetics and can provide indispensable means for young researchers to become principal investigators and future European leaders by integrating them into networks of experienced scientists/clinicians. EC funding schemes should devote further effort to principal investigators career development.

The European Union is currently funding over 300 epigenetics projects (a High Impact Project, Collaborative Projects, Networks of Excellence, ERC (European Research Council) Starting Grants, ERC Advanced Grants, Marie Curie Actions) with a total contribution of more than €200 Million.

## Abbreviations

EC-COST: European Commission's Cooperation in Science and Technology
DG RTD: European Commission's Directorate General for Research and Innovation
DNA: Deoxyribonucleic acid
EZH2: Enhancer of zeste homolog 2
US FDA: United States Food and Drug Administration
MDS: Myelodysplastic Syndrome
CTCL: Primary Cutaneous T-cell Lymphoma
nc: Non-coding
RNAs: Ribonucleic acids
EU: European Union
ERC: European Research Council.
